# Impact of an emergency medical dispatch system on survival from out-of-hospital cardiac arrest: a population-based study

**DOI:** 10.1186/s13049-016-0247-y

**Published:** 2016-04-22

**Authors:** François-Xavier Ageron, Guillaume Debaty, Angèle Gayet-Ageron, Loïc Belle, Arnaud Gaillard, Marie-France Monnet, Stéphane Bare, Jean-Christophe Richard, Vincent Danel, Jean-Pierre Perfus, Dominique Savary

**Affiliations:** Department of Emergency Medicine - SAMU 74, Annecy Genevois Hospital, Annecy, France; Northern French Alps Emergency Network, Department of Public Health, Annecy Genevois Hospital, Annecy, France; Department of Emergency Medicine, University Hospital of Grenoble, Grenoble, France; Division of Clinical Epidemiology, Department of Health and Community Medicine, University of Geneva Hospitals, Geneva, Switzerland; Department of Cardiology, Annecy Genevois Hospital, Annecy, France; Haute-Savoie Fire Department, Meythet, France; Isère Fire Department, Fontaine, France; Department of Emergency Medicine - SAMU 73, Saint-Jean de Maurienne Hospital, Saint-Jean de Maurienne, France

**Keywords:** Dispatch centre, Out-of-hospital cardiac arrest, Emergency phone number, Cardiopulmonary resuscitation, Survival

## Abstract

**Background:**

In countries where a single public emergency telephone number is not in operation, different emergency telephone numbers corresponding to multiple dispatch centres (police, fire, emergency medical service) may create confusion for the population about the most appropriate service to call. In particular, out-of-hospital cardiac arrest (OHCA) requires a prompt and effective response. We compare two different dispatch systems on OHCA patient survival at 30 days in a national system with multiple emergency telephone numbers.

**Methods:**

We conducted an observational retrospective study of 6871 patients aged 18 years or older with presumed OHCA of cardiac origin between 2005 and 2013 in three counties of the Northern French Alps region. One county had a single dispatch centre combining medical and fire emergencies, and two had multiple dispatch centres. Propensity score matching analyses were performed to compare patient survival at 30 days.

**Results:**

A total of 2257 emergency calls for OHCA were managed by a single dispatch centre and 4614 by a multiple dispatch centre. A single dispatch centre was associated with an increase in survival (adjusted odds ratio [OR] for all patients: 1.7; 95 % confidence interval [CI] = 1.3–2.2; *p* <0.001; adjusted OR for propensity-matched patients: 2.0; 95 % CI = 1.2–3.4; *p* = 0.012).

**Conclusions:**

A single dispatch centre was associated with a markedly improved increase of survival among OHCA patients at 30 days in a system with several emergency telephone numbers.

**Electronic supplementary material:**

The online version of this article (doi:10.1186/s13049-016-0247-y) contains supplementary material, which is available to authorized users.

## Background

Out-of-hospital cardiac arrest (OHCA) is a leading cause of death worldwide [1]. Successful resuscitation of these patients requires a coordinated set of rescuer actions termed the “chain of survival” [2]. An early call to the emergency medical service (EMS) and the recognition of cardiac arrest by the dispatcher is the first link in this chain [3]. The recognition of cardiac arrest may be challenging, but it is an essential skill to provide appropriate advice for cardiopulmonary resuscitation (CPR) and to activate a rapid response by the EMS [4]. In many countries worldwide, specific emergency telephone numbers are available to the population. In North America, the 9–1–1 number is the public emergency number to contact the police, medical and fire services [5]. By contrast, in other regions, particularly in South America, Africa and Europe, there is often no single public emergency number and several national emergency telephone numbers are available with one dispatch centre for each service. In the case of OHCA, the fire department and EMS are both mainly the first responders on the scene and this could lead to some confusion for the population as to which telephone number is the most appropriate to dial [6]. The European Union has implemented a unique emergency telephone number, but only a few countries and regions have adopted it as their sole public emergency number with a single dispatch function.

In the Northern French Alps region, the two systems of emergency dispatch co-exist. We hypothesized that patient survival following OHCA may differ depending on the emergency dispatch system in use. The aim of this observational prospective study was to compare the association between a single and multiple dispatch centre and survival among adults with OHCA.

## Methods

### Dispatch systems in France and the Northern French Alps

The French national telephone number 1–5 corresponds to the EMS dispatch centre in charge of medical emergencies and is staffed by emergency physicians. The national telephone number 1–8 corresponds to the fire department dispatch centre. The European Union emergency number 1–1–2 was introduced in 2002 [7], but in most member states, it remains an additional number to national emergency-specific numbers. In France, 1–1–2 calls are answered by the fire department in most regions. The French health care system for emergencies has been described previously [8]. In brief, it is a two-tier system comprising a basic life support fire department ambulance and an advanced life support physician-staffed EMS ambulance. Each service dispatches its own ambulance in coordination with the other dispatch centre. Two ambulances (advanced life support and basic life support) are sent out for all OHCA.

**Fig. 1:**
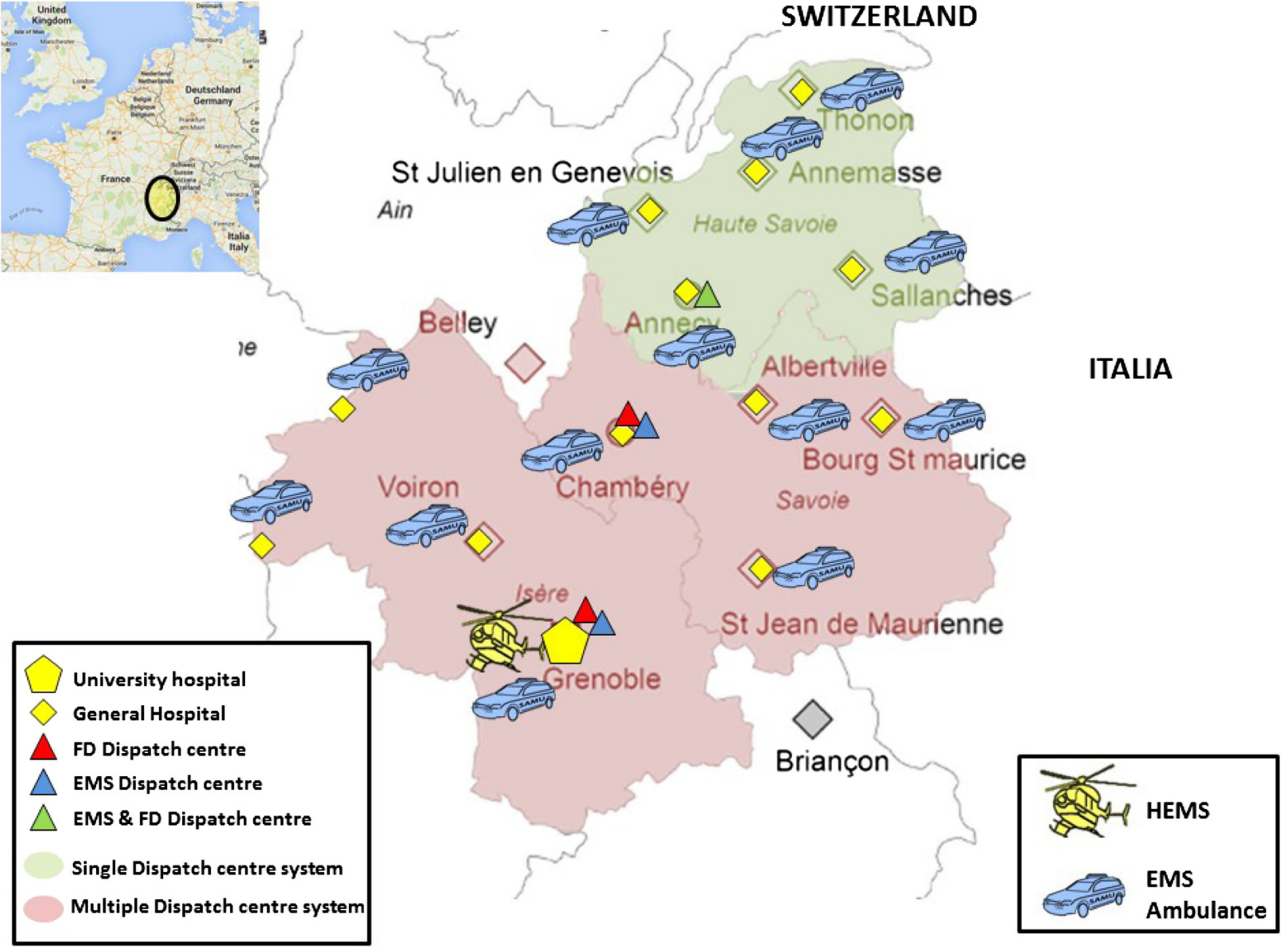
Emergency medical service system in the Northern French Alps Emergency Network. In the area with the single dispatch centre system, there are 92 BLS ambulances in 89 rescue centres for a population of 782,900 inhabitants in 4388 km2. In the area with the multiple dispatch centre system, there are 216 BLS ambulances in 208 rescue centres for a population of 1,556,600 inhabitants in 13,459 km2. The number of ambulances per inhabitant : 11.8 ambulances/100.000 inhabitants in the single dispatch centre system and 13.9 ambulances/100.000 inhabitants in the multiple dispatch centre system. BLS ambulance are evenly distributed to cover the entire area with a maximum on-site time of 20 min.BLS: basic life support; FD: fire department; EMS: emergency medical service

**Fig. 2:**
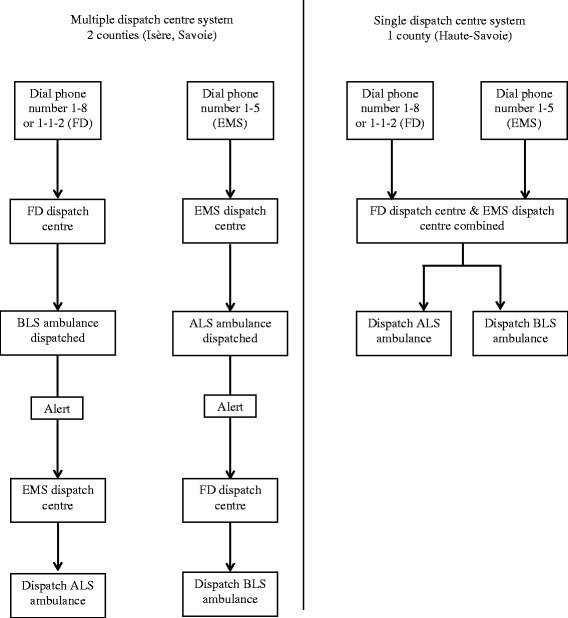
Pathway of emergency calls for out-of-hospital cardiac arrest depending on the dispatch centre system in the Northern French Alps. FD: fire department; EMS: emergency medical service; BLS: basic life support; ALS: Advanced life support

In the three counties of the Northern French Alps (Isère, Savoie and Haute-Savoie), two follow the national system described above with multiple dispatch centres (Fig. 1). An emergency physician is present only in the EMS dispatch centre. Since 1995, the third county has implemented a single dispatch centre, which answers 1–1–2, 1–5 and 1–8 calls. The pathway for an emergency call in each dispatch centre system is shown in Fig. 2.

### Study design and patients

This was a retrospective observational cohort study of all patients presenting with OHCA in the Northern French Alps region between 1 January 2005 and 31 December 2013. Only cases of OHCA of presumed cardiac etiology were selected. We excluded OHCA without attempted resuscitation. Patients with trauma, including drowning and strangulation/hanging, respiratory disease, haemorrhage, and those less than 18 years old were excluded. The study complied with the Declaration of Helsinki and was approved by the ethics committee of the University Hospital of Clermont Ferrand, Clermont Ferrand, France.

### Cardiac arrest registry data collection

The study is based on patient data collected by the Northern French Alps Cardiac Arrest Registry. All patients for whom an ambulance was dispatched to start CPR following OHCA are included in the registry. The Northern French Alps Emergency Network comprises all hospitals and EMS systems in the three counties, including all other rescue services (fire department, police and army), and represents a region of 18,000 km^2^ with a population of 2 million inhabitants. The network elaborates the regional protocol regarding acute coronary disease and cardiac arrests and organizes several meetings each year with the participation of many emergency physicians. Since 1 January 2005, each case of OHCA is recorded in an electronic form completed by the emergency physician in charge of the patient and the dispatch centre. The form includes relevant variables, such as age, place of OHCA, presence of a witness, CPR, initial cardiac rhythm recorded, presumed etiology, defibrillation, and intervention times. Survival at hospital discharge and at 30 days is collected by research technicians in charge of collecting quality control data.

### Primary outcome

The primary endpoint was survival at 30 days.

### Statistical analysis

We compared the characteristics of patients managed by single *vs*. multiple dispatch centres. Depending on the application criteria, continuous variables were compared using either Student’s t-test or the Mann–Whitney U test and categorical variables were compared using the Chi-2 test or Fisher’s exact test. The total incidence rate of cardiac arrest and the incidence rate of the selected population in the different geographic zones were estimated in order to effect a comparison by univariate analysis; the global survival rate in each area was compared using the Chi-2 test.

We then performed a multivariate analysis to assess the association between survival and the type of the dispatch centre system, adjusted on the usual predictors of survival after cardiac arrest. First, a logistic regression model was used with survival at 30 days as a dependent variable. Independent variables were a single dispatch centre (*vs*. multiple dispatch center), age, gender, emergency telephone number used (1–5 *vs*. 1–8/1–1–2), presence of witness, place of cardiac arrest (home *vs*. public place), first documented cardiac rhythm (shockable vs non shockable rhythm), period of international guidelines on cardiac arrest (years 2005–2010 *vs*. 2011–2013), and initial admission to a catheterization laboratory. Second, as patients were not randomly assigned to each group, we performed a propensity score matching analysis to limit this selection bias. We calculated the propensity score, reflecting the probability that a patient would be assigned to a single dispatch *vs.* a multiple dispatch centre based on all the independent variables that had no potential effect related to the single/multiple dispatch centres. Using the Stata macro programme *psmatch2*, patients in the single dispatch centre group were matched with a unique patient in the multiple dispatch centre group with the same demographics and cardiac arrest characteristics using the nearest neighbour methods of the propensity score. After matching a selection of patients, we performed a conditional regression model to assess the association between the survival rate and dispatch centre system.

Interactions of the multivariate models were tested when they were plausible (interaction between single/multiple dispatch centres and the first call to 1–8 fire department or 1–5 EMS numbers) using a likelihood ratio test and we kept those having *p* < 0.10. The goodness of fit of the model was tested using the Hosmer-Lemeshow test and the predictive value of the model was assessed by post-estimation receiver operating characteristic curves. To investigate the effect of the use of a single dispatch centre on the patient survival rate potentially mediated by a third variable, we performed causal mediation analysis using parametric regression models [9]. The two-sided significance level was *p* < 0.05. All statistics were performed using Stata SE, version 11.0 (StataCorp, College Station, TX, USA).

## Results

**Fig. 3:**
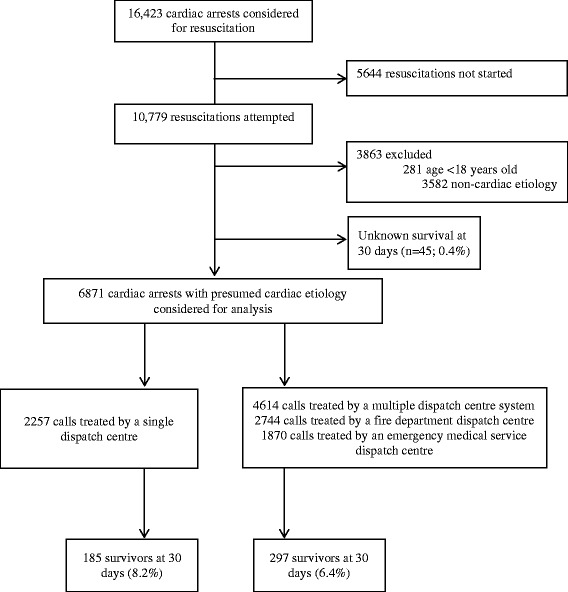
Study flow chart based on the Northern French Alps Cardiac Arrest Registry, 2005–2013

Between 1 January 2005 and 31 December 2013, 16,423 OHCA cases considered for resuscitation were recorded in the registry. Among these, 6871 patients were considered for further analysis (Fig. 3). A total of 2257 emergency calls were managed by a single dispatch centre and 4614 by a multiple dispatch centre. The EMS telephone number 1–5 was called first for 2894 (42 %) OHCA cases. Patient characteristics are summarized in Table 1. The presence of witnesses was significantly lower in the single dispatch centre group. Time from first call to arrival on the scene was significantly shorter in the single dispatch centre group. The time from the first call to the first attempted CPR was reduced in the single dispatch centre group compared to the multiple dispatch centre group (median time, 5 min and 8 min, respectively; *p* < 0.001). The proportion of bystander CPR was significantly higher in the single dispatch centre group. Table 2 summarizes the overall population-based results according to the different dispatch systems. The incidence of OHCA for which an ambulance was dispatched to start CPR was 78/100,000 inhabitants/year.

**Table 1:** Patient characteristics at the time of out-of-hospital cardiac arrest according to single or multiple dispatch centre, 2005–2013: Northern French Alps Cardiac Arrest Registry (*n =* 6871)

Characteristics	Single dispatch centre (*n =* 2257)	Multiple dispatch centre (*n =* 4614)	*Test value*	*p*-value
First call to, n (%)			14.5	<0.001
1–8/1–1–2 FD emergency number	1233 (54.6)	2744 (59.5)		
1–5 EMS emergency number	1024 (45.4)	1870 (40.5)		
Age, mean (SD), y	69 (15)	68 (15)	–2.6	0.008
Male, n (%)	1655 (73.3)	3274 (71.0)	4.3	0.038
Witness, n (%)	1666 (73.8)	3701 (80.2)	32.3	<0.001
Bystander witness	1355 (60.0)	3137 (68.0)		
EMS witness	311 (13.8)	564 (12.2)		
CPR by bystander witness, n (%)	693 (30.7)	1270 (27.5)	7.5	0.006
Cardiac arrest occurred at home, n (%)	1618 (71.7)	3417 (74.1)	10.5	0.015
Shockable rhythm, n(%)	656 (29.1)	1233 (26.7)	94.7	<0.001
Median time in min from call to				
Arrival on scene	10 [7–14]	11 [8–16]	10.8	<0.001
First CPR attempted	5 [0–11]	8 [0–14]	6.5	<0.001
First shock attempted	12 [7–18]	14 [9–19]	2.9	0.035
Initial admission to cath lab, n (%)	115 (20.8)	257 (28.8)	11.7	0.001
Any ROSC, n (%)	705 (31.3)	1055 (22.9)	55.7	<0.001
Hospital admission alive, n (%)	554 (24.6)	891 (19.3)	25.0	<0.001

Statistical tests used were: Student T test for age, Mann–Whitney U test for times from call to arrival on scene, to fisrt CPR attempted, to first shock. Chi-2 test was used for the others variables

*FD* fire department, *EMS* emergency medical service, *CPR* cardiopulmonary resuscitation, *SD* standard deviation, *IQR* interquartile range, *Cath lab* catheterization laboratory, *ROSC* return of spontaneous circulation

**Table 2:** Overall incidence of cardiac arrest in the population-based study according to the different dispatch systems, 2005–2013: Northern French Alps Cardiac Arrest Registry

	Total No (N/100,000/year)	Single dispatch centre system No (N/100,000/year)	Multiple dispatch centre system No (N/100,000/year)	*p*-value
Overall population	2,339,616	782,924	1,556,692	
Cardiac arrest included in the registry	16,424 (78)	5630 (80)	10,794 (77)	0.03
Cardiac arrest considered for resuscitation	10,779 (52)	3775 (54)	7004 (50)	<0.001
Cardiac arrest included in the study	6871 (33)	2257 (32)	4614 (33)	0.28
30 day-survival of OHCA included in the study	482 (2.3)	185 (2.6)	297 (2.1)	0.02

OHCA: out-of-hospital cardiac arrest

Using crude data, survival at 30 days was 1.8 % higher when a single dispatch centre was used compared to a multiple dispatch centre (*p* = 0.007). When adjusted for all potential confounding factors in multiple logistic regression or propensity score models, we confirmed that survival at 30 days was significantly higher when OHCA was managed through a single dispatch centre (Table 3). Depending on the models, survival at 30 days was higher by 70 % (multiple logistic regression) to 100 % (propensity score model) with the single dispatch centre system compared to the multiple dispatch centre.

**Table 3:** Survival at 30 days of patients with out-of-hospital cardiac arrest according to type of dispatch centre, 2005–2013; Northern French Alps Cardiac Arrest Registry (*n =* 6871)

	Single dispatch centre system		Multiple dispatch centre system						
	No Total	No (%) Survivors	No Total	No (%) Survivors	ARR (95 % CI)	Unadjusted OR (95 % CI)^a^	*p-*value	Adjusted OR (95 % CI)^a^	*p- value*
30-day survival (*n =* 6871)	2257	185 (8.2)	4614	297 (6.4)	+1.8 (0.4–3.1)^b^	1.30 (1.07–1.57)	0.007	1.70 (1.30–2.22) ^d^	<0.001
30-day survival in propensity score matching analysis (*n =* 4510)^a^	2255	185 (8.2)	2255	140 (6.3)	+1.9 (0.4–3.4)^b^	1.32 (1.05–1.66)	0.016	2.00 (1.16–3.43) ^e^	0.012

*CI* confidence interval, *ARR* absolute risk reduction, *OR* odds ratio, *cath lab* catheterization laboratory

^a^Indicates OR for single dispatch system compared to multiple dispatch centre system (reference OR = 1.00)

^b^Indicates greater survival for single dispatch centre system

^c^Propensity score matching analysis based on covariates at the first call (ventricular fibrillation, age, sex, place of cardiac arrest, witness to collapse, first call to fire department telephone number, period of cardiac arrest (2005–2010 and 2011–2013), cath lab first admission (*n =* 6869). 2255 patients in single dispatch group and 2255 patients in multiple dispatch group

^d^Unconditional logistic regression adjusted for covariates at the first call (ventricular fibrillation, age, sex, place of cardiac arrest, witness to collapse, first call to fire department telephone number, period of cardiac arrest [2005–2010 and 2011–2013], cath lab first admission) (*n =* 6866). Logistic regression = 1672; *p* <0.001; R2 = 0.48; area under the curve = 0.92; 95 % CI, 0.91–0.94

^e^Conditional logistic regression based on propensity score matching analysis and adjusted on covariates at the first call (ventricular fibrillation, age, sex, place of cardiac arrest, witness to collapse, period of cardiac arrest [2005–2010 and 2011–2013], cath lab first admission (*n =* 4510). Logistic regression = 320; *p* < 0.001; R2 = 0.76; area uder the curve = 0.86; 95 % CI, 0.84–0.89

There was a significant interaction between a single *vs*. a multiple dispatch centre system and the first call dialled by witnesses (EMS *vs*. fire department telephone number) (*p* = 0.027). Table 4 reports stratum-specific estimates of the association between survival at 30 days and the dispatch centre system by the first dialled emergency telephone number. Witnesses who called the fire department phone number 1–8/1–1–2 through the multiple dispatch centre system were only managed by the fire department dispatch centre and this was associated with a worse outcome with an odds ratio of survival at 30 days of 0.44 (*p* < 0.001). By contrast, witnesses who dialled first the fire department telephone number through the single dispatch centre were systematically transferred to an emergency physician and this was associated with a greater increase of survival at 30 days by 2.25–fold (*p* < 0.001) in this subgroup. Of note, survival at 30 days was unchanged when 1–8 was dialled first compared to when 1–5 was dialled first in the single dispatch centre (*p* = 0.314). Survival at 30 days was also unchanged when 1–5 was dialled first (*p* = 0.270) in the single dispatch centre compared to the multiple dispatch centre. The effect of the single dispatch centre on the patient survival rate was partially mediated by the response time (Additional file 1).

**Table 4:** Stratum-specific estimate of the association between the dispatch centre system and telephone number dialled and survival outcome at 30 days

	30-day survival adjusted OR (95 % CI)^a^	*p*-value
FD telephone number 1–8 dialled first compared to EMS telephone number 1–5 dialled first		
In the multiple dispatch centre system (i.e., FD dispatch centre alone *vs*. EMS dispatch centre alone)	0.44 (0.32–0.63)^b^	0.001
In the single dispatch centre system	0.81 (0.53–1.22)^b^	0.314
Single dispatch centre system compared to the multiple dispatch centre system		
When EMS telephone number 1–5 is dialled first (i.e., single dispatch centre *vs*. EMS dispatch centre alone)	1.24 (0.84–2.21)^c^	0.270
When FD telephone number 1–8 is dialled first (i.e., single dispatch centre *vs.* FD dispatch centre alone)	2.25 (1.56–3.24)^c^	<0.001

*EMS* emergency medical service, *FD* fire department, *OR* odds ratio, *CI* confidence interval; cath lab: catheterization laboratory

^a^Unconditional logistic regression adjusted for covariates at the first call (ventricular fibrillation, age, sex, place of cardiac arrest, witness to collapse, first call to FD telephone number, period of cardiac arrest [2005–2010 and 2011–2013], cath lab first admission) (*n =* 6866). Logistic regression = 1672; *p* <0.001; R2 = 0.48; area under the curve = 0.92; 95 % CI, 0.91–0.94

^b^Indicates OR for FD phone number compared to EMS phone number (reference OR = 1.00)

^c^Indicates OR for single dispatch compared to the multiple dispatch centre (reference OR = 1.00)

## Discussion

Our findings indicate that OHCA managed through a single dispatch centre compared to a multiple dispatch centre is independently associated with an increase of survival at 30 days. Poor outcome associated with 1–8/1–1–2 fire department telephone numbers in the multiple dispatch centre system was not confirmed in the single dispatch centre system. This supports the effectiveness of the single dispatch centre, particularly when individuals dialled the fire department telephone number. No statistically significant effect was found when individuals dialled the EMS 1–5 number first in a single vs multiple dispatch centres.

To our knowledge, this is the first study to report an association between survival and the type of emergency dispatch system included in the survival chain. Several studies have demonstrated the benefit of implementing specific procedures in emergency dispatching, such as the “medical priority dispatch” protocol [10–12], but none has formally demonstrated the link between the emergency organization and chance of survival. The emergency organization in our geographical region is probably uncommon as it is rare to find different dispatch systems in the same area. This specific organization allowed to compare two dispatch systems coexisting in a homogenous population, which could limit selection and classification bias. A German study showed that only 20 % of OHCA witnesses dialled the EMS telephone number, thus demonstrating a confusion in the population as to the appropriate emergency number to use [13]. In a system with several emergency numbers, Lipps and colleagues [6] reported that only 14 % of emergency calls reached the EMS directly, thus representing a time-consuming transfer of calls from one dispatch centre to another and less time for resuscitation. Finland is one of the rare European countries that has decided to use only the 1–1–2 telephone number connected to the different dispatch centres. Maatta and colleagues [14] observed that the fusion of existing centres increased call answering and processing times, but did not affect the accuracy of dispatching decisions. In another Finnish study, Lindstrom and colleagues reported that after the reform of the emergency medical communication system, dispatchers tended to underestimate the priority of the call compared to the period before the reform [15]. Following difficulties to evaluate the dispatch center effectiveness, performance indicators had to be set up according to organisational consideration. However, none of these studies assessed the effect of these systems on survival.

Our results show that the response times to the first attempted CPR and the arrival of an ambulance on the scene were shorter in the single dispatch centre compared to the multiple dispatch centre. This could be linked to the time taken by the EMS dispatcher to activate the chain of survival by requesting the fire department dispatcher in a distant centre to send out a basic life support ambulance. The bystander CPR rate was also higher in the single dispatch centre group. The fact that all calls were centralized to an EMS dispatcher and, in most cases, to emergency physicians in a single dispatch centre, may explain the higher bystander CPR rate through a better recognition of cardiac arrest and by supporting bystander CPR by telephone. In France, the training of EMS dispatchers requires specific skills defined by the ministry of health. By contrast, the training of fire department dispatchers is not supported or defined by health authorities. Thus, differences in skill levels could explain the significant difference in patient survival when individuals dialled first the 1–8/1–1-2 fire department number. A single dispatch centre combines the rapidity of fire department first responders with the medical capacity of the EMS to detect the presence of OHCA and to provide CPR instructions to the caller.

As shown in different studies, the early recognition of cardiac arrest and the decrease of time to CPR are key elements to improve survival. Hollenberg and colleagues [16] showed that by dispatching EMS and fire department responders in parallel, the time from first call to arrival on the scene significantly decreased and survival significantly improved from 5.7 % to 9.7 % in the case of OHCA. Joslyn and colleagues [17] reported that the existence of a single emergency telephone number, such as 9–1–1, improved survival and decreased the time to first shock in OHCA. In our study, we were able to link the benefit of survival observed in the single dispatch centre group to the reduced response time. We observed that first responders arrived 3 min earlier in the single dispatch centre group and the time for defibrillation was reduced by a median of 2 min in in the same group. As each minute of delay to defibrillation educes the probability of survival to discharge by 10–12 %, we could argue that a part of the benefit observed is due to a shortened response time [18]. In a study based on the Stavanger cardiac arrest registry, Lindner and colleagues highlighted that survival increased from 18 % to 25 % when all parts of the survival chain were improved [19]. The correct identification of cardiac arrest requires well-trained dispatchers [20]. Berdowski and colleagues [4] showed that a lack of training increased the time for ambulance dispatching and the time from the call to arrival on the scene. In the case of non-identification of OHCA, survival was 5 % vs 14 %. Kuisma and colleagues [21] reported that survival improvement was linked to the number of opportunities to receive emergency calls for cardiac arrest; the more calls received by the dispatcher, the higher the probability of survival. Dispatchers delivering instructions for CPR to witnesses are included in international guidelines for the management of cardiac arrest and some studies have shown the effectiveness of this recommendation [22–25].

Our study has some limitations. First, calls were not randomly assigned to single or multiple dispatch centres, but based on the geographical area concerned. We tried to minimize confusion and selection bias by the use of a propensity score with matching of patients using the nearest neighbour method, but we were unable to control for all unknown confounders as in a randomized controlled trial. However, it would be extremely difficult to conduct such a trial in the context of comparing two systems in different geographical areas. Second, we excluded OHCA of non-cardiac etiology from our analysis as these patients usually present a worse outcome. Therefore, we chose to restrict our study population in order to investigate the effectiveness of the organization of the dispatch systems. Moreover, we analysed the results of the entire OHCA population and found similar results (data not presented). Third, a cluster effect could represent another limitation. Training of pre-hospital and hospital teams may vary between centres and might explain differences in outcome. However, the regional tertiary referral hospital is located in one of the multiple dispatch centre counties and cannot explain the survival difference observed. Finally, we cannot establish a causal link between the increase of survival and the structural differences in the two dispatch systems. Even if the population and the prehospital systems seem comparable, other factors may exist in the EMS system that contribute to this difference.

## Conclusion

These results support the effectiveness of a single dispatch centre for OHCA management, which integrates emergency physicians in the survival chain, thereby increasing the chance of patient survival by an appropriate and rapid response time. If the population is confused about the appropriate telephone number to dial for medical emergencies, a single dispatch centre could represent an alternative to a public emergency number.

## Additional file

Additional file 1: Association between dispatch center type and outcomes of survival at 30 days according to response time (logistic regression with causal mediation model). (DOCX 13 kb)

## References

[1] Berdowski J, Berg RA, Tijssen JG, Koster RW (2010). Global incidences of out-of-hospital cardiac arrest and survival rates: Systematic review of 67 prospective studies. Resuscitation.

[2] Cummins RO, Ornato JP, Thies WH, Pepe PE (1991). Improving survival from sudden cardiac arrest: the “chain of survival” concept. A statement for health professionals from the Advanced Cardiac Life Support Subcommittee and the Emergency Cardiac Care Committee, American Heart Association. Circulation.

[3] Hazinski MF, Nolan JP, Billi JE, Bottiger BW, Bossaert L, de Caen AR (2010). Part 1: Executive summary: 2010 International Consensus on Cardiopulmonary Resuscitation and Emergency Cardiovascular Care Science With Treatment Recommendations. Circulation.

[4] Berdowski J, Beekhuis F, Zwinderman AH, Tijssen JG, Koster RW (2009). Importance of the first link: description and recognition of an out-of-hospital cardiac arrest in an emergency call. Circulation.

[5] Davis DP, Garberson LA, Andrusiek DL, Hostler D, Daya M, Pirrallo R (2007). A descriptive analysis of Emergency Medical Service Systems participating in the Resuscitation Outcomes Consortium (ROC) network. Prehosp Emerg Care.

[6] Lipp M, Mihaljevic V, Dick W (1994). Analysis of requests for help. Emergency calls to the fire department and emergency medical services, as well as to the general medical practitioners’ emergency services in a rescue service area. Anaesthesist.

[7] European Council (2012). Implementation of the European emergency number 112 – Results of the fifth data-gathering round, Ed Brussels.

[8] Adnet F, Lapostolle F (2004). International EMS systems: France. Resuscitation.

[9] VanderWeele TJ (2013). A three-way decomposition of a total effect into direct, indirect, and interactive effects. Epidemiology.

[10] Heward A, Damiani M, Hartley-Sharpe C (2004). Does the use of the Advanced Medical Priority Dispatch System affect cardiac arrest detection?. Emerg Med J.

[11] Nurmi J, Pettila V, Biber B, Kuisma M, Komulainen R, Castren M (2006). Effect of protocol compliance to cardiac arrest identification by emergency medical dispatchers. Resuscitation.

[12] Weiser C, van Tulder R, Stockl M, Schober A, Herkner H, Chwojka CC et al. Dispatchers impression plus Medical Priority Dispatch System reduced dispatch centre times in cases of out of hospital cardiac arrest. Pre-alert - A prospective, cluster randomized trial. Resuscitation. 2013;84(7):883-8. 10.1016/j.resuscitation.2012.12.017 23295777

[13] Diehl P, Mauer D, Schneider T, Dick W (1992). The emergency telephone number--the essential weak link in an emergency system. Prospective studies involving cardiac arrests observed by bystanders. Anaesthesist.

[14] Maatta T, Kuisma M, Vayrynen T, Nousila-Wiik M, Porthan K, Boyd J (2010). Fusion of dispatching centres into one entity: effects on performance. Acta Anaesthesiol Scand.

[15] Lindstrom V, Pappinen J, Falk AC, Castren M (2011). Implementation of a new emergency medical communication centre organization in Finland--an evaluation, with performance indicators. Scand J Trauma Resusc Emerg Med.

[16] Hollenberg J, Riva G, Bohm K, Nordberg P, Larsen R, Herlitz J (2009). Dual dispatch early defibrillation in out-of-hospital cardiac arrest: the SALSA-pilot. Eur Heart J.

[17] Joslyn SA, Pomrehn PR, Brown DD (1993). Survival from out-of-hospital cardiac arrest: effects of patient age and presence of 911 Emergency Medical Services phone access. Am J Emerg Med.

[18] Perkins GD, Handley AJ, Koster RW, Castren M, Smyth MA, Olasveengen T (2015). European Resuscitation Council Guidelines for Resuscitation 2015: Section 2. Adult basic life support and automated external defibrillation. Resuscitation.

[19] Lindner TW, Soreide E, Nilsen OB, Torunn MW, Lossius HM (2011). Good outcome in every fourth resuscitation attempt is achievable--an Utstein template report from the Stavanger region. Resuscitation.

[20] Calle PA, Lagaert L, Vanhaute O, Buylaert WA (1997). Do victims of an out-of-hospital cardiac arrest benefit from a training program for emergency medical dispatchers?. Resuscitation.

[21] Kuisma M, Boyd J, Vayrynen T, Repo J, Nousila-Wiik M, Holmstrom P (2005). Emergency call processing and survival from out-of-hospital ventricular fibrillation. Resuscitation.

[22] Svensson L, Bohm K, Castren M, Pettersson H, Engerstrom L, Herlitz J (2010). Compression-only CPR or standard CPR in out-of-hospital cardiac arrest. N Engl J Med.

[23] Kellermann AL, Hackman BB, Somes G (1989). Dispatcher-assisted cardiopulmonary resuscitation. Validation of efficacy. Circulation.

[24] Rea TD, Eisenberg MS, Culley LL, Becker L (2001). Dispatcher-assisted cardiopulmonary resuscitation and survival in cardiac arrest. Circulation.

[25] Sayre MR, Koster RW, Botha M, Cave DM, Cudnik MT, Handley AJ (2010). Part 5: Adult basic life support: 2010 International Consensus on Cardiopulmonary Resuscitation and Emergency Cardiovascular Care Science With Treatment Recommendations. Circulation.

