# Supplementary material for: Impact of an emergency medical dispatch system on survival from out-of-hospital cardiac arrest: a population-based study
Source: Scand J Trauma Resusc Emerg Med. 2016 Apr 22;24:53. doi: 10.1186/s13049-016-0247-y (PMC4840865; doi:10.1186/s13049-016-0247-y)
Supplement: Additional file 1: — Association between dispatch center type and outcomes of survival at 30 days according to response time (logistic regression with causal mediation model). (DOCX 13 kb) [file 13049_2016_247_MOESM1_ESM.docx]

**Additional table 1.** Association between dispatch center type and outcomes of survival at 30 days according to response time (logistic regression with causal mediation model)

| Single dispatch center and mediation variables | Survival at 30 days | *p*-value | PM % |
| --- | --- | --- | --- |
|  | OR (95% CI) ^a^ |  |  |
| Time from call to arrival on scene ^b^ |  |  |  |
| Natural direct effect | 1.7 (1.2-2.4) | .004 |  |
| Natural indirect effect | 1.1 (1.0-1.2) | .005 |  |
| Total effect | 1.9 (1.3-2.7) | <.001 | 23 |
| Time from call to first CPR attempted ^c^ |  |  |  |
| Natural direct effect | 1.5 (0.7-3.2) | .288 |  |
| Natural indirect effect | 1.2 (1.1-1.3) | <.001 |  |
| Total effect | 1.8 (0.9-3.9) | .117 | 39 |

OR: odds ratio; PM: proportion mediated

^a^ Total effect indicates the global effect on survival of the single dispatch center compared to multiple dispatch center. Total effect is decomposed in two Odds ratio. The natural direct effect corresponds to the effect of the single dispatch centre itself (without mediation variable). And the natural direct effect corresponds to the effect of the single dispatch centre due to the response time (time from call to arrival on scene or to first CPR attempted).

^b^ Parametric multivariate regression model 1 with causal mediation (mediated variable: time from call to arrival on scene) and the following covariates (age, gender, place of cardiac arrest, initial rhythm recorded, witness, period of guideline, initial catheterization laboratory admission), n=5948.

^c^ Parametric multivariate regression model 2 with causal mediation (mediated variable: time from call to first CPR attempt) and the following covariates (age, gender, place of cardiac arrest, initial rhythm recorded, witness, period of guideline, initial catheterization laboratory admission), n=6829.
